# Calibration Model Optimization for Strain Metrology of Equal Strength Beams Using Deflection Measurements

**DOI:** 10.3390/s23063059

**Published:** 2023-03-13

**Authors:** Yonggang Yan, Zhengxing Wu, Jianjun Cui, Kai Chen, Yanhong Tang, Ning Yang

**Affiliations:** 1 School of Mechanical and Power Engineering, Henan Polytechnic University, Jiaozuo 454003, China; 2 National Institute of Metrology, Beijing 100029, China; 3 Metrology and Testing Institute of Tibet Autonomous Region, Lhasa 850000, China; 4 Shaanxi Institute of Metrology Science, Xi’an 710100, China

**Keywords:** metrology, calibration model, strain sensor, equal strength beam, deflection measurement

## Abstract

Strain sensors, especially fiber Bragg grating (FBG) sensors, are of great importance in structural health monitoring, mechanical property analysis, and so on. Their metrological accuracy is typically evaluated by equal strength beams. The traditional strain calibration model using the equal strength beams was built based on an approximation method by small deformation theory. However, its measurement accuracy would be decreased while the beams are under the large deformation condition or under high temperature environments. For this reason, an optimized strain calibration model is developed for equal strength beams based on the deflection method. By combining the structural parameters of a specific equal strength beam and finite element analysis method, a correction coefficient is introduced into the traditional model, and an accurate application-oriented optimization formula is obtained for specific projects. The determination method of optimal deflection measurement position is also presented to further improve the strain calibration accuracy by error analysis of the deflection measurement system. Strain calibration experiments of the equal strength beam were carried out, and the error introduced by the calibration device can be reduced from 10 με to less than 1 με. Experimental results show that the optimized strain calibration model and the optimum deflection measurement position can be employed successfully under large deformation conditions, and the deformation measurement accuracy is improved greatly. This study is helpful to effectively establish metrological traceability for strain sensors and furthermore improve the measurement accuracy of strain sensors in practical engineering scenarious.

## 1. Introduction

Strain measurement is of great significance in structural health monitoring, mechanical property analysis, and so on, and is taken commonly by means of all kinds of strain sensors, such as resistance strain gauges and fiber optic strain gauges. Of those, fiber Bragg grating (FBG) sensors are frequently utilized because of many advantages, including high accuracy, fast response, electrical passivity, corrosion resistance, low-cost production, and wide use in aerospace [1], transportation [2], engineering and construction [3], and marine applications [4]. The measurement accuracy of the strain sensors will be restricted by high-temperature environments, packaging types, and working layouts. So, it is very essential to precisely evaluate the metrological characteristics of these sensors using a novel calibration method and a standard device. At present, the commonly-used strain calibration devices are the four-point bending device [5,6], universal testing machine [7], and equal strength beam. However, the first two devices have some disadvantages of complex operation, large scale, and difficult metrological traceability. 

As a high-precision elastic sensitive component, equal strength beams have many advantages, such as good mechanical properties, high accuracy, and simple operation, and are often used in the study and calibration of vibration sensors [8], displacement sensors [9,10] and strain sensors [11,12,13,14]. In particular, the metrological calibration of FBG strain sensors with the strain and temperature coupling effects [15], can be possibly carried out using equal strength beams. Nowadays, the calibration model based on an approximate method is employed while strain sensors are measured by means of equal strength beams. It is very difficult to precisely evaluate the metrological performance of strain sensors and to establish a strain metrology system.

Currently, the widely used strain calibration methods mainly include the load method and the deflection method using equal strength beams in engineering. The former is supposed to calculate the strain on the surface of an equal strength beam based on the elastic modulus, end load, and structural size of the beam [16,17,18]. Zhu et al. [19] hanged weights at the end of the equal strength beam, calculated the surface strain of the beam according to the ending loads, and then calibrated the distributed fiber optic strain sensor affixed to the surface of the beam. Huang et al. [20] also used the load method to investigate the performance of the resistance strain gauge, FBG strain sensor, and wireless strain sensor by calculating the surface strain of the equal strength beam. However, there exist some problems, such as incorrect control of the mass and the continuity of the applied load, and the imprecise characteristic parameters of the beam, such as elastic modulus in the calculation process. Therefore, it is difficult to implement the high-precision metrological traceability of the strain sensor. To solve this problem, some researchers used the deflection method to calibrate strain sensors. The deflection method is used to calculate the surface strain of an equal strength beam based on the deflection of the beam [21,22,23]. Tu et al. [24] used the method to calculate the surface strain of an equal strength beam, finding that there was a good linear relationship between the strain value and the load. Hou et al. [25] calibrated the FBG strain sensor and evaluated the uncertainty of the calibration results using the method. The relation of the deflection and strain of the equal strength beam was established successfully, and the high precision metrological traceability of FBG sensors was realized. 

Although the deflection method has avoided the problem of inaccurate values for the elastic modulus of equal strength beams, it still has some problems, such as low accuracy and ambiguous measurement positions. The commonly used calculation model of deflection method is an approximate formula based on the theory of small deformations of beams in the material mechanics [26]. Polilov et al. [27] proposed that the theory of small deformations can only be used for calculations at the time when the first order derivative term of the deflection is so small as to be negligible. However, it is difficult to find the critical point of deformation accurately in the actual calibration of strain sensors, and the small deformation condition can hardly be met. As for the large deformation theory of the beams, many studies have started to investigate and have made some achievements [28,29,30]. However, due to the non-linearity of the mathematical model [31,32], it is difficult to apply the method to practical engineering, with complicated calculations and low efficiency. To complete the metrological traceability of strain sensors under complex conditions, the strain calibration model and the deflection measurement position of the equal strength beams are indispensable to improve the accuracy of the strain metrology calibration.

In this paper, the deflection method strain calibration model is discussed and simulated, and an optimization model is proposed. The simulation comparison between the optimized model and the traditional model verifies the effectiveness. Then, the factors affecting the accuracy of strain measurement in the practical application are analyzed, and the optimum deflection measurement position is proposed. Finally, the proposed optimization model and the optimal deflection measurement point are verified by experiments, and the results demonstrate that both can effectively improve the accuracy of the surface strain calculation for the equal strength beam. Therefore, the optimized method can effectively improve the accuracy of strain sensors in practical applications. In addition, it will also provide a solid foundation for the establishment of a strain metrology system.

## 2. Novel Calibration Model and Method

### 2.1. Theoretical Analysis

**Figure 1:**
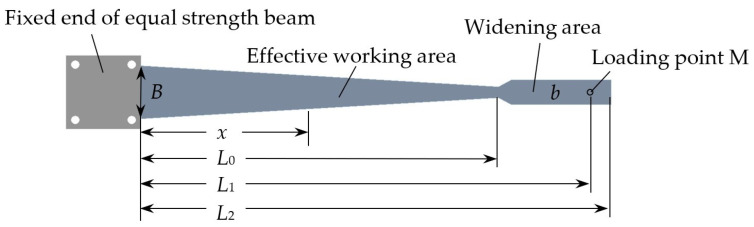
Structure of the equal strength beam. In the figure, *B* is the width of the effective working area at the fixed end; *b* is the width of the widening area; *x* is the distance from any point on the beam to the fixed end; *L*_0_ is the effective length; *L*_1_ is the working length; *L*_2_ is the total length; The thickness of the whole beam is *h*.

A commonly used equal strength beam structure is shown in Figure 1, consisting of a fixed end, an effective working area, and a widening area. The effective working area refers to the permissible adhesion area of the strain sensor under test. The widening area is used to bear external loads.

According to the knowledge of material mechanics, when a load of *P* is applied to the loading point M, the strain generated on the surface of the beam can be expressed as [33]:

(1) $$\varepsilon =\frac{\sigma }{E}=\frac{6PL_{1}}{EBh^{2}}$$

where E is the elastic modulus of the equal strength beam, and is a constant related to its material. σ is the normal surface stress.

In practice, the elastic modulus E is often taken imprecisely due to material characteristics. To avoid the introduction of strain calculation error, the deflection is used to calculate the strain on the surface of the beam. The differential equation for the deflection curve of an equal strength beam is:

(2) $$\frac{\frac{d^{2}w}{dx^{2}}}{{\left[1+{\left(\frac{dw}{dx}\right)}^{2}\right]}^{\frac{3}{2}}}=\frac{M\left(x\right)}{EI\left(x\right)}$$

where *w* is the deflection of the equal strength beam at *x*, *M(x)* is the bending moment at *x*; *I(x)* is the cross-section moment of inertia at *x*, expressed as:

(3) $$I(x)=\frac{b(x)h^{3}}{12}=\frac{Bh^{3}(L_{1}-x)}{12L_{1}}$$

where *b(x)* is the width of the equal strength beam at *x*.

For small deformations at the end of the beam, i.e., d*w*/d*x* ≪ 1, Equation (2) can be simplified to:

(4) $$\frac{d^{2}w}{dx^{2}}=\frac{M(x)}{EI(x)}$$

Integrating Equation (4) twice, and according to the boundary condition that the slope and deflection are zero at the fixed end of the beam, it can be obtained:

(5) $$w=\frac{6PL_{1}}{EBh^{3}}x^{2}$$

combining Equations (1) and (5), the expression is given as follows:

(6) $$\varepsilon =\frac{h}{x^{2}}w$$

It can be seen from Equation (6) that the surface strain ε of the equal strength beam can be calculated by measuring the thickness *h*, deflection *w* and deflection measurement position *x*. This strain calibration model is simple and easy to be applied in engineering. However, Equation (6) is a simplified formula derived from Equation (2). Only when the equal strength beam has small deformation can the accuracy of surface strain measurement be guaranteed. When the deformation of the beam is large, the strain measurement accuracy decreases so seriously that the accurate metrological traceability of the strain sensor cannot be achieved precisely.

To improve the accuracy of strain metrological traceability while retaining the advantages of the simplicity of the deflection method, a finite element method was used to analyze the strain of equal strength beam, and the optimization method of strain metrology calibration model was established.

### 2.2. Optimization of the Deflection Method Strain Calibration Model 

#### 2.2.1. Strain Simulation of Equal Strength Beam

The commonly used equal strength beam is taken as an example, and its specific parameters are shown in Table 1 and Table 2.

**Figure 2:**
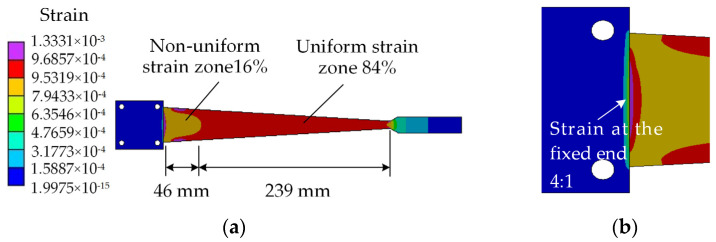
(**a**) Strain distribution on the surface of the beam with a load of 100 N; (**b**) Partial enlarged view of the edge of the fixed end.

The three-dimensional model of the equal strength beam was established and was imported into Ansys Workbench to mesh by the finite element analysis software. Here, the size of the element was set to 2 mm, and then the strain simulation analysis was carried out. According to the material properties of the beam and the allowable stress, a load of 100 N was applied to the loading point at the end of the beam. The strain distribution cloud diagram is shown in Figure 2a. The simulation results show that uniform and non-uniform strain zones are generated in the effective working area on the beam surface, and the strain in the uniform strain zone was approximately 961 με. 

It can be seen from Figure 2a that the percentage of uniform strain zone is to 84% in the effective working area of the beam. Certainly, there exists non-uniform strain zone near the fixed end, which is inconsistent with the ideal performance of the equal strength beam. Seen from Figure 2b, strain has also occurred at the edge of the fixed end of the beam. It shows that local deformations are caused by the non-linearity of the beam structure and have an effect on the mechanical properties of the equal strength beam. That is to say, the non-linearity performance leads to the generation of the non-uniform strain zone. In addition, there is also a non-uniform strain area near the widening area. Therefore, the strain sensor should be affixed to the uniform strain zone in the effective working area when calibrating the strain sensor.

In order to verify the accuracy of the simulation results, the strain simulation values in the uniform strain zone (*x* = 172 mm) of the equal strength beam with different loads were extracted, and the theoretical strain values were calculated by Equation (1), as shown in Table 3.

The maximum relative error between the theoretical and simulated values is 0.230%, and the difference is about 1 με. The comparison results show that the simulation results are significantly consistent with the theoretical results. Therefore, the simulation results of the equal strength beam are used as the reference value in order to modify the simplified Equation (6).

#### 2.2.2. Optimization of the Strain Calculation Model 

By the total differentiation of Equation (6), we can get:

(7) $$\Delta \varepsilon =\frac{h}{x^{2}}\Delta w-\frac{2hw}{x^{3}}\Delta x$$

**Figure 3:**
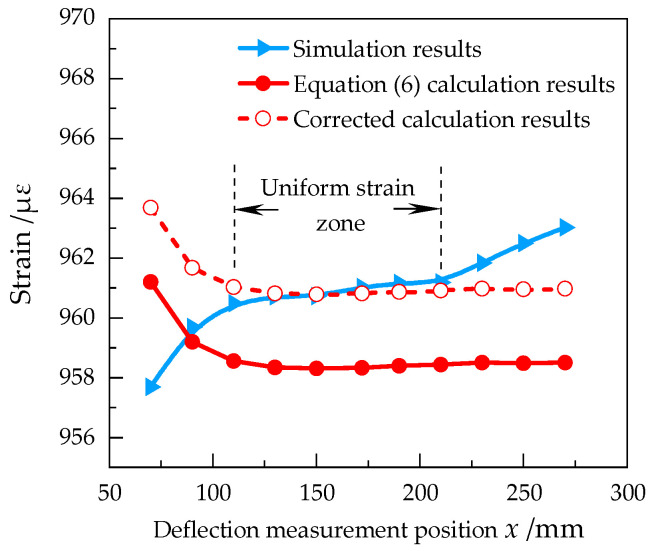
The strain simulation results at different positions with 100 N load, the calculation results of Equation (6), and the corrected calculation results of Equation (6).

It can be seen from Equation (7) that the strain error is closely related to the variation of deflection and the position of deflection. Therefore, the simulated strain and deflection values were taken at different positions within the effective working area. Here, the values were sampled by the equal intervals based on the simulation results with a load of 100 N (see Figure 2), and the strain values were also calculated by Equation (6). The results are shown in Figure 3.

As can be seen from Figure 3, there are still non-uniform strains in the simulation results curve, so it is determined that the uniform strain zone of the equal strength beam is within the range of *x* = 110~210 mm. Moreover, the strain fluctuation within the range is no more than 1 με. Within the uniform strain zone, the calculated strain value is obviously different from the simulated value. The calculation results curve is not ideally straight, with rapid changes in the front and smoother changes in the middle and end. It was indicated that it was consistent with the theoretical guidance results.

Considering the metrological performance evaluation of the strain sensor, the smooth segment of the calculation results curve can be corrected to correspond to the uniform region of the simulation results curve. The difference was relatively constant between the calculated value and the simulated value in the uniform strain zone, and the average deviation of each measurement position can be used for the correction coefficient. The corrected curve was shown in Figure 3. After the correction, the average difference between the calculated strain value and the simulated strain value decreases from −2.49 με to −0.01 με in the uniform strain zone, as shown in Table 4.

**Figure 4:**
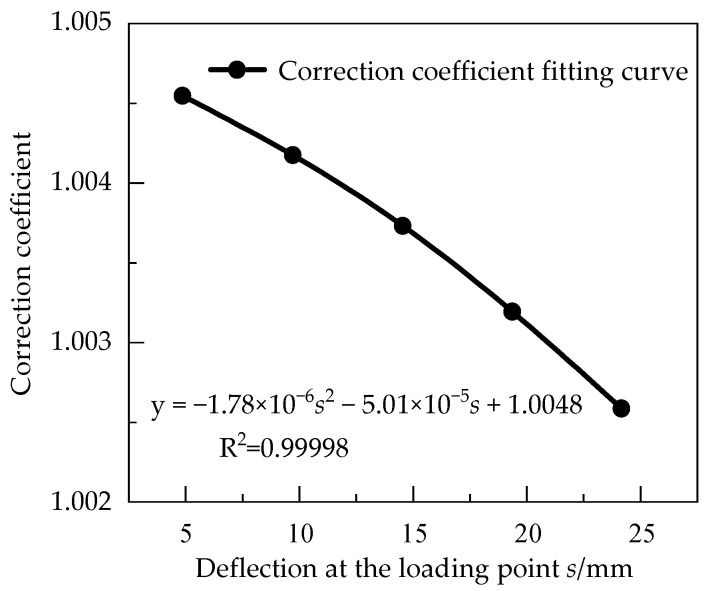
Correction coefficient fitting curves for different deflections at the loading point.

According to Equation (7), the strain calculation error varied with the deflection. In order to correct the calculated value of strain with different strains, loads of 20 N, 40 N, 60 N and 80 N were applied at the loading point respectively. The strains 193 με, 386 με, 579 με, and 770 με were produced on the surface of the equal strength beam and the deflection at the loading point corresponding to each strain value was recorded. Meanwhile, the strain values were also calculated by Equation (6). The strain calculation results are corrected according to the correction method under a load of 100 N. Finally, the scatter plot of the correction coefficient varying with the deflection at the loading point with different strains is obtained and fitted with a quadratic polynomial, as shown in Figure 4.

Therefore, the relation between the correction coefficient K and the deflection *w* of the loading point is set as:

(8) $$K\left(s\right)=aw^{2}+bw+c$$

According to the fitting results in Figure 4, a = −1.78 × 10^−6^, b = −5.01 × 10^−5^, c = 1.0048. The goodness of fit coefficient R^2^ is 0.99998, and the results show that the strain correction coefficient presents a quadratic function variation rule.

By introducing Equation (8) into Equation (6), we can get:

(9) $$\varepsilon =\frac{h}{x^{2}}w\cdot K\left(s\right)$$

#### 2.2.3. Simulation Result

**Figure 5:**
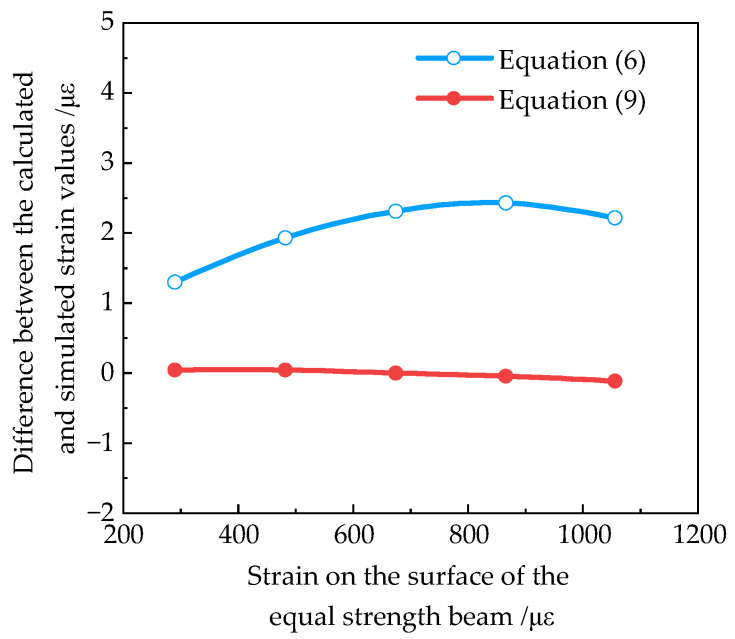
Difference between calculated and simulated values of Equations (6) and (9).

Equation (9) is the optimized strain calculation formula of the deflection method. In order to verify the validity of Equation (9), the load of 30 N, 50 N, 70 N, 90 N, and 110 N was applied respectively at the loading point in the simulation, and the corresponding strains on the surface of the equal strength beam are 290 με, 482 με, 674 με, 865 με, and 1056 με, respectively. The difference between the calculated and simulated strain values obtained by Equations (6) and (9), respectively, at a certain position in the uniform strain zone (*x* = 130 mm) is shown in Figure 5.

It can be seen from Figure 5 that the corrected deviation at different strains is less than 0.2 με. It was indicated that Equation (9) can effectively improve the accuracy of theoretical strain calculation. At the same time, it can be seen that when the strain of the equal strength beam is below 300 με, an accurate strain value can also be obtained by using Equation (6).

## 3. Determination of Deflection Measurement Position 

### 3.1. Error Analysis of Deflection Measurement

**Figure 6:**
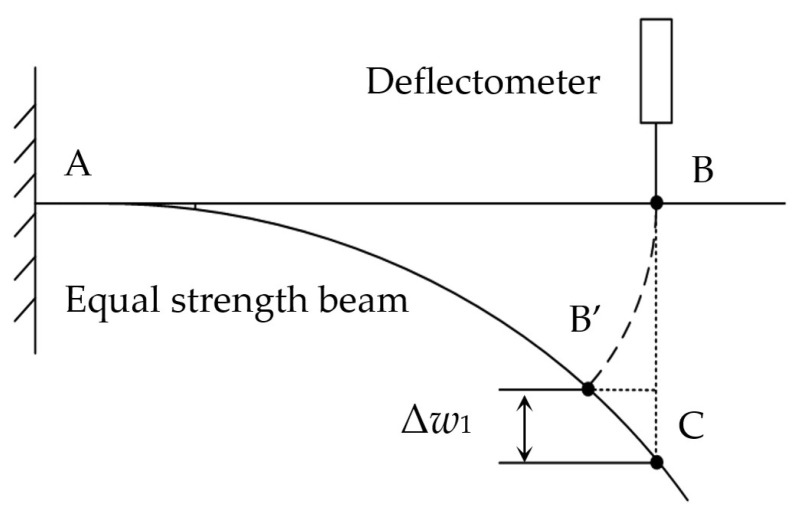
Deflection measurement position offset.

When the deflection method is used to measure the strain of the equal strength beam, the deflection measurement will introduce errors in addition to the principal errors under large deformation condition. When one kind of deflectometer is used to measure the deflection of an equal strength beam, the position of the deflectometer is usually fixed. The axial displacement, caused by the beam bending, will change the measurement position of the deflection. As shown in Figure 6, the deflection measurement position changes from point B to C after the beam was bent. If the change of deflection measuring position is not taken into account during strain calculation, a deflection measurement error of Δ*w*_1_ will be introduced, and make the calculated strain value larger. The corresponding error was called the measurement position offset error and was denoted as +Δ*ε*_p_.

**Figure 7:**
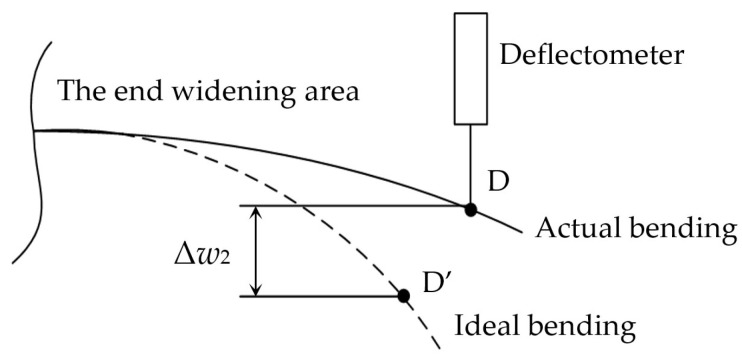
Deflection measurement error in the end widening area of an equal strength beam.

Ideally, the equal strength beam has an isosceles triangle structure. It is necessary to apply the concentrated loads at the triangle vertices so as to ensure a uniform strain zone on the surface of the equal strength beam [34]. However, due to the limitations of the load application conditions, the vertex of the triangle is often widened practically to facilitate the application of load (see Figure 1), but it also changes the bending ability of the end of the equal strength beam. As shown in Figure 7, the bending degree of the widening area is smaller than the ideal after the beam is bent with the load. At the time, the measured deflection of the widening area is smaller than the theoretical deflection. This will introduce the deflection measurement error of Δ*w*_2_ and make the calculated strain value smaller. The corresponding error is called the end measurement error and is denoted as −Δ*ε*_r_.

**Figure 8:**
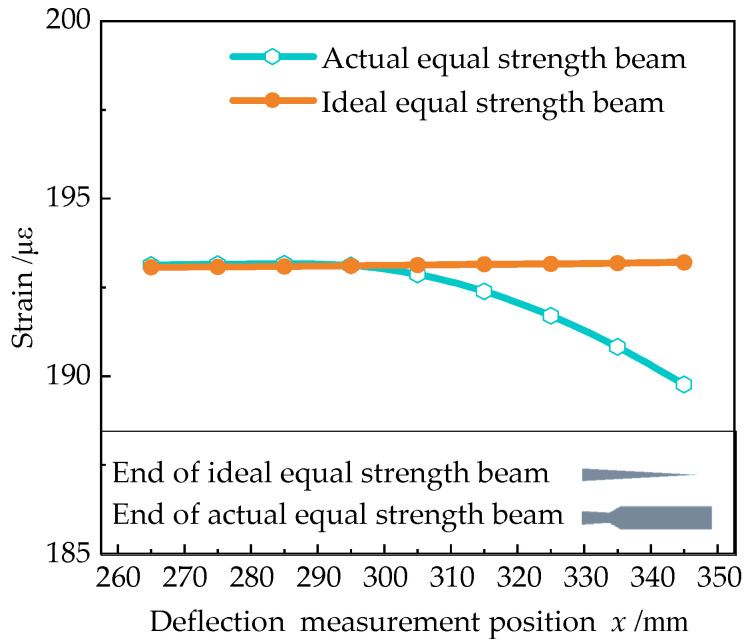
Comparison of calculated strain values at the end of an ideal and actual equal strength beam.

To further observe the end measurement error, the ideal beam and the actual were numerically simulated in Ansys Workbench. A fixed load was applied at the loading point, and the deflection at different positions was taken at equal intervals at the end of the beam. The strain value was calculated by Equation (9). The results were shown in Figure 8. The result shows that the calculated strain value at the end of the actual equal strength beam is smaller than that of the ideal, i.e., the end measurement error is introduced.

According to the analysis in this section, the measurement position offset error +Δ*ε*_p_ will make the calculated strain value larger, and the end measurement error −Δ*ε*_r_ will make the calculated strain value smaller. Therefore, if the deflectometer is fixed at the end of the equal strength beam for measurement, there will be an optimum deflection measurement position. Thus, the strain measurement error is minimal with the condition of |+Δ*ε*_p_| = |−Δ*ε*_r_|.

### 3.2. Numerical Simulation Analysis 

**Figure 9:**
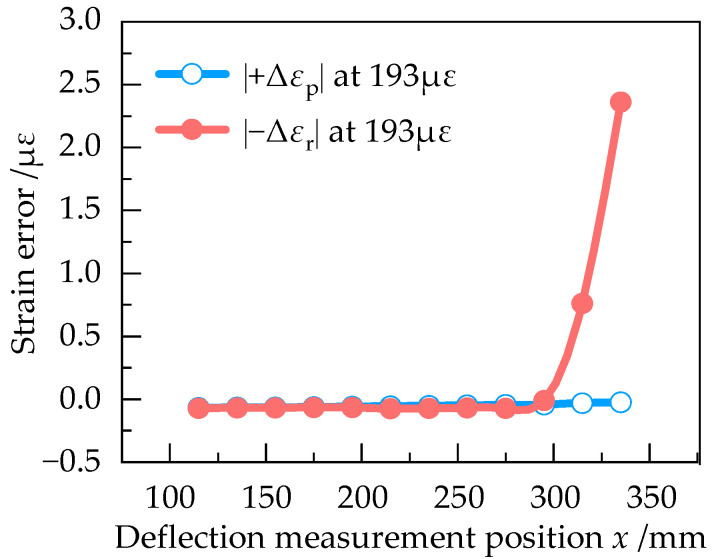
Curve of |+Δε_p_| and |−Δε_r_| at 193 με.

**Figure 10:**
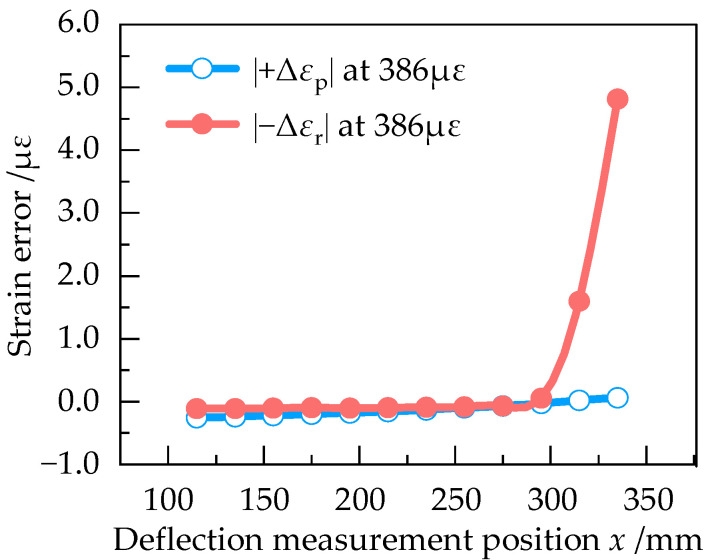
Curve of |+Δε_p_| and |−Δε_r_| at 386 με.

**Figure 11:**
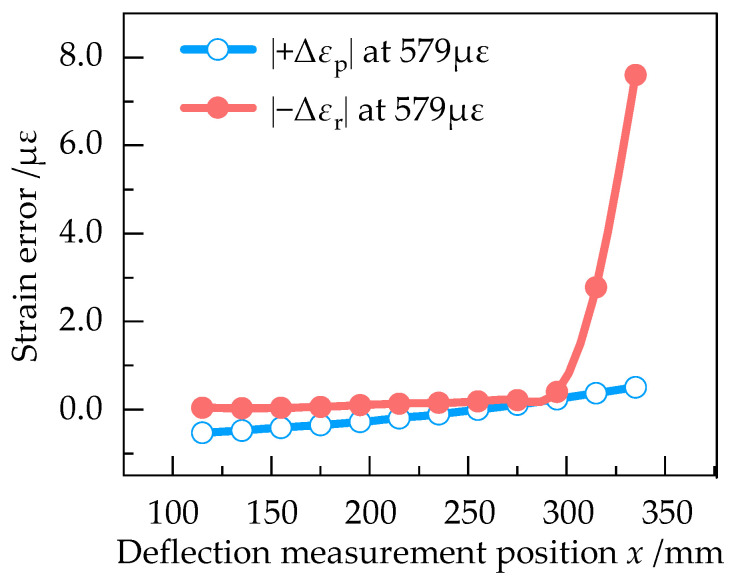
Curve of |+Δε_p_| and |−Δε_r_| at 579 με.

**Figure 12:**
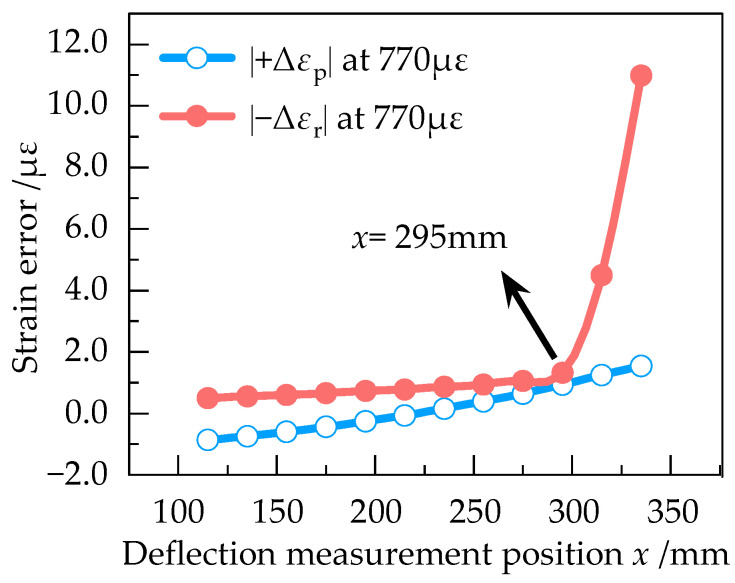
Curve of |+Δε_p_| and |−Δε_r_| at 770 με.

**Figure 13:**
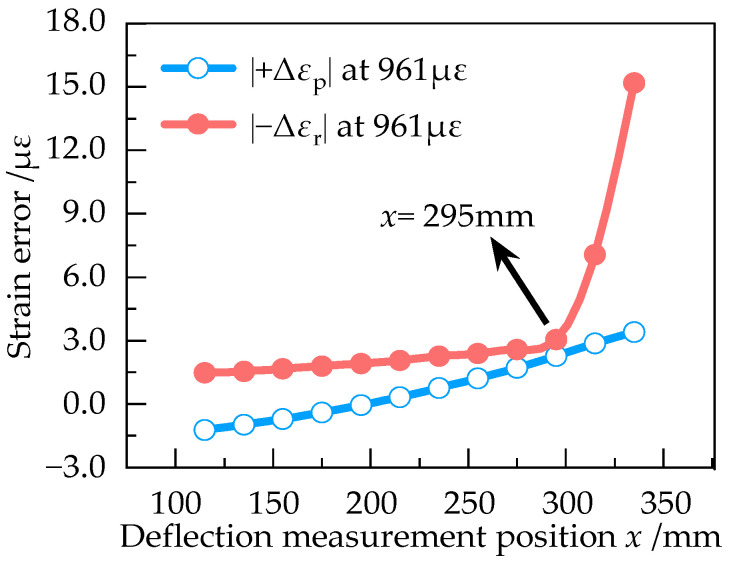
Curve of |+Δε_p_| and |−Δε_r_| at 961 με.

In order to determine the optimum deflection measurement position, |+Δ*ε*_p_| and |−Δ*ε*_r_| at different positions of the equal strength beam with the strain of 193 με, 386 με, 579 με, 770 με and 961 με were numerically simulated in Ansys Workbench. The results are shown in Figure 9, Figure 10, Figure 11, Figure 12 and Figure 13.

According to Figure 9, |+Δ*ε*_p_| and |−Δ*ε*_r_| are approximately equal at 193 με in the effective working area, indicating that the measurement error is small when the strain of equal strength beam is small. From Figure 9, Figure 10, Figure 11, Figure 12 and Figure 13, it can be seen that when the strain increases from 193 με to 961 με, |+Δ*ε*_p_| and |−Δ*ε*_r_| always keep a small difference at *x* = 295 mm. Therefore, *x* = 295 mm is the optimum deflection measurement position of this equal strength beam, located at the junction of the effective working area and the widening area.

## 4. Experiments

### 4.1. Experimental Setup

**Figure 14:**
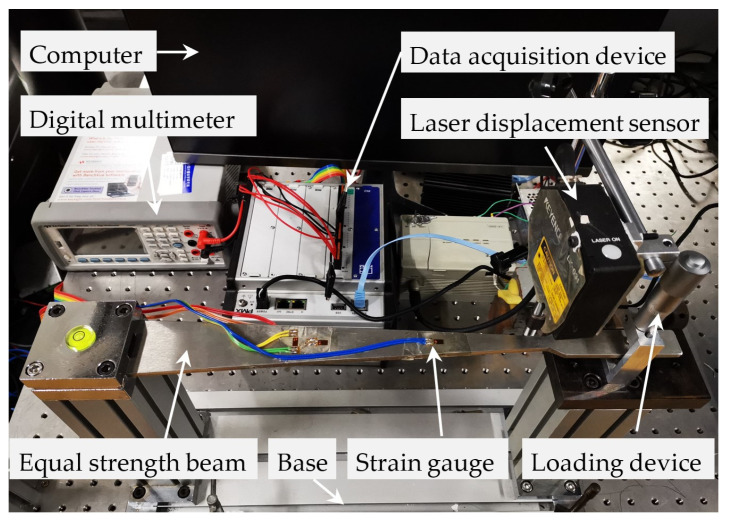
Experimental setup for the strain measurement of the equal strength beam.

In order to verify the correctness and validity of the optimized strain calculation model for the deflection method and the optimum deflection measurement position for equal strength beams, a strain measurement experiment was carried out using the equal strength beam and resistance strain gauge. The experiment layout is shown in Figure 14. The equal strength beam was fixed horizontally to a stable base, and its dimensional parameters are shown in Table 1. The loading device was also fixed on the base, with the same position as the loading point of the equal strength beam. It was used to apply a load to the equal strength beam. The resistance strain gauges (initial resistance: 120.51 Ω, accuracy: ±0.1 Ω, gauge factor: 2.18) were axially attached to the surface of the equal strength beam to measure the strain produced by the beam. The strain measurement data were collected by the data acquisition device (HBM, sampling frequency: 2400 Hz, resolution: 0.1 με) connected to the resistance strain gauges and displayed on the computer. The laser displacement sensor (KEYENCE, LK-031, sensitivity: 1.000943 V/mm, accuracy: ±0.006 mm) was fixed above the equal strength beam to measure the deflection of the beam. The deflection measurement data were collected by a digital multimeter (KEYSIGHT, resolution bits: 7^1/2^) and stored on the computer. The ambient temperature of the experiment was 20 ± 0.1 °C to reduce the influence of temperature on the resistance strain gauge.

### 4.2. Strain Measurement Experiment

The laser displacement sensor was fixed at *x* = 295 mm. The load was applied at the loading point M (see Figure 1) of the equal strength beam in steps of 1.5 mm from 0 to 10.5 mm. The corresponding result of the resistance strain gauge measurement was 0–407.75 με. The measurement data of the laser displacement sensor and resistance strain gauges were recorded during the loading process. 

#### 4.2.1. Validation of the Optimized Strain Calculation Formula

Equations (6) and (9) were used to calculate the strain of the equal strength beam, and the differences between the calculated results and the measurement results of resistance strain gauges are shown in Table 5.

As can be seen from Table 5, the deviation of Equation (6) increased as the deflection of the loading point increased, while the deviation of Equation (9) remained around 0 με, which was consistent with the simulation results (see Figure 5). This indicated that the optimized formula could effectively reduce the strain calculation error.

#### 4.2.2. Influence of Deflection Measurement Position on Strain Calculation Results

According to the numerical simulation analysis, the optimum deflection measurement position was at the junction of the effective working area and the widening area of the equal strength beam. Therefore, multiple positions were selected at small intervals around the widening area and individual positions were selected at large intervals in the effective working area. The position *x* of the laser displacement sensor was changed sequentially to 140, 160, 180, 240, 260, 285, 290, 300, 305, and 310 mm, and loaded again after each change. The strain of the equal strength beam was calculated by Equation (9), and the calculated results were compared with the measured results of resistance strain gauges. 

**Figure 15:**
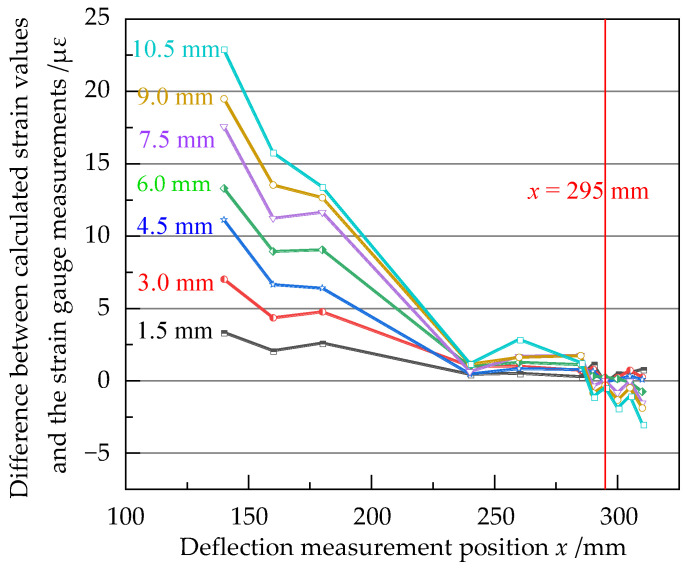
Difference between calculated strain values and the strain gauge measurements at the different deflection measurement positions under each loading step.

The measured results of the resistance strain gauge were used as a reference, and the calculated result of Equation (9) was subtracted from them to obtain the difference. The variation of the difference with the deflection measurement position under different loads is shown in Figure 15.

As can be seen from Figure 15, as the deflection measurement position *x* was changed from 140 to 310 mm, the difference under each load was generally on the decrease. In the range of 140~240 mm, when the deflection of the loading point increased from 1.5 to 10.5 mm, the difference increased significantly, and the maximum difference exceeded 10 με. In the range of 240~310 mm, the variation of the difference was less pronounced. Here, at *x* = 295 mm, the difference was kept at around 0 με under each load, which was better than the other measurement positions. Therefore, *x* = 295 mm was the optimum deflection measurement position of this equal strength beam, and this position was at the junction of the effective working area and the widening area, which was consistent with the simulation results. After *x* = 295 mm, the difference started to increase slowly. It could be found that the strain calculation error was smaller when the deflection was measured close to the junction of the effective working area and the widening area, and larger when it was far away.

## 5. Discussion

In the experimental results, the strain measurement error can be reduced from 10 με to less than 1 με in the range of 407.75 με, effectively improving the strain measurement accuracy. The simulation and experimental results show that the differences between the optimized calculation results and the measured results of the reference resistance strain gauges remain around 0 με with the increase of load. The optimization model can improve the strain calculation accuracy compared with the traditional calculation formula and solve the limitations of the traditional. The model can be suitable for the strain measurement for large deformation, and it is convenient for practical engineering application. In the paper, the authors do not take the temperature into consider. However, it is no doubt that the presented strain measurement model of the beam should be improved if the beam is used to calibrate strain sensors, especially FBG sensors, under high temperature. Hence, the strain calibration model will be studied under the load and temperature in the future.

Moreover, the results of the study provide a reference for the selection of deflection measurement locations for the deflection method. According to the principle of the deflection method, the measurement position can be chosen arbitrarily, and the strain can then be calculated from the deflection. However, the end of an actual equal strength beam is often widened, and the deflection of the widened area is smaller than the ideal deflection. On the other hand, the beam usually has a lateral displacement during bending, which leads to a deflection measurement point shift and introduces measurement errors. Therefore, the position of deflection measurement has a non-negligible influence on the calculation result of strain. To meet the need of strain metrology, the optimal deflection measurement position has been found correctly. It can improve the accuracy of strain calculations for equal strength beams. When the deflection is measured by the deflectometer at the optimum deflection position, the difference remains around 0 με with the increase of the load between the strain calculation result and the reference resistance strain gauge. This indicates that the position is better than the others, and the determination method of the optimal deflection measurement position is suitable in the calibration using the beam. 

The optimum deflection measurement position is at the junction of the effective working area and the widening area of the equal strength beam. It can be found that the strain measurement error is smaller when the deflection is measured close to the junction, and larger when it is far away. This result provides a useful reference for the selection of deflection measurement positions in practical applications.

## 6. Conclusions

In this paper, an optimized strain calculation model and the determination method of the optimal deflection measurement position were developed based on the deflection method to effectively improve the strain calibration accuracy using the equal strength beams under large deformation conditions. To improve the strain calibration accuracy of equal strength beams at large deflections, a correction coefficient was introduced into the conventional deflection method strain calibration model by finite element analysis, and an optimized strain calculation formula was obtained. Error analysis of the deflection measurement system was carried out to find the optimum deflection measurement position. A comparison experiment with the traditional model was conducted, and the results prove that the proposed strain measurement method of the equal strength beam is valid and corrective. 

In further research, this study will be applied to the calibration of optical fiber sensors in normal or high-temperature environments. Additionally, the effect of the thermal expansion of equal strength beams on strain calibration will also be investigated. This study not only provides a high-accuracy strain metrology standard, but also gives a basis for the selection of the deflection measurement position in the strain measurement of equal strength beams.

## Figures and Tables

**Table 1:** Physical dimensions of the equal strength beam.

Dimensions	Symbols	Numerical Values/mm
Thickness	*h*	5.0
Width at the fixed end	*B*	43.2
Width of the widening area	*B*	20.0
Effective length	*L*_0_	285.0
Working length	*L*_1_	360.0
Total length	*L*_2_	375.0

**Table 2:** Material parameters of the equal strength beam.

Material	Density	Elastic Modulus	Poisson’s Ratio
65 Mn	7850 kg/m^3^	2.08 × 10^5^ Mpa	0.26

**Table 3:** Comparison of simulated and theoretical values with different loads.

No.	Applied Load/N	Theoretical Value/με	Simulation Value/με	Relative Error
1	40	384.62	385.82	0.052%
2	60	576.92	578.25	0.230%
3	80	769.23	770.06	0.108%
4	100	961.54	961.02	−0.054%

**Table 4:** Strain difference before and after correction with a load of 100 N.

No.	Measurement Position *x*/mm	Difference before Correction/με	Difference after Correction/με
1	110	−1.93	0.55
2	130	−2.38	0.10
3	150	−2.42	0.06
4	172	−2.68	−0.20
5	190	−2.77	−0.29
6	210	−2.74	−0.27
	Average	−2.49	−0.01

**Table 5:** Differences between strain gage measurements and calculated results in Equations (6) and (9).

Deflection of Loading Point M/mm	Deviation of Equation (6)/με	Deviation of Equation (9)/με
0.0	0.00	0.00
1.5	0.37	0.14
3.0	0.50	0.01
4.5	0.85	0.10
6.0	0.74	−0.26
7.5	1.22	−0.02
9.0	1.76	0.31
10.5	2.11	0.47

## Data Availability

Not applicable.
